# Supplementary material for: Comprehensive Annotation and Functional Exploration of MicroRNAs in Lettuce
Source: Front Plant Sci. 2021 Dec 24;12:781836. doi: 10.3389/fpls.2021.781836 (PMC8739914; doi:10.3389/fpls.2021.781836)
Supplement: Supplementary file 2 [file Data_Sheet_8.ZIP › GSM2230749.plot/Lsa-miRN3_Lsat_1_v5_gn_1_73301.1_3178_TPlot.pdf]

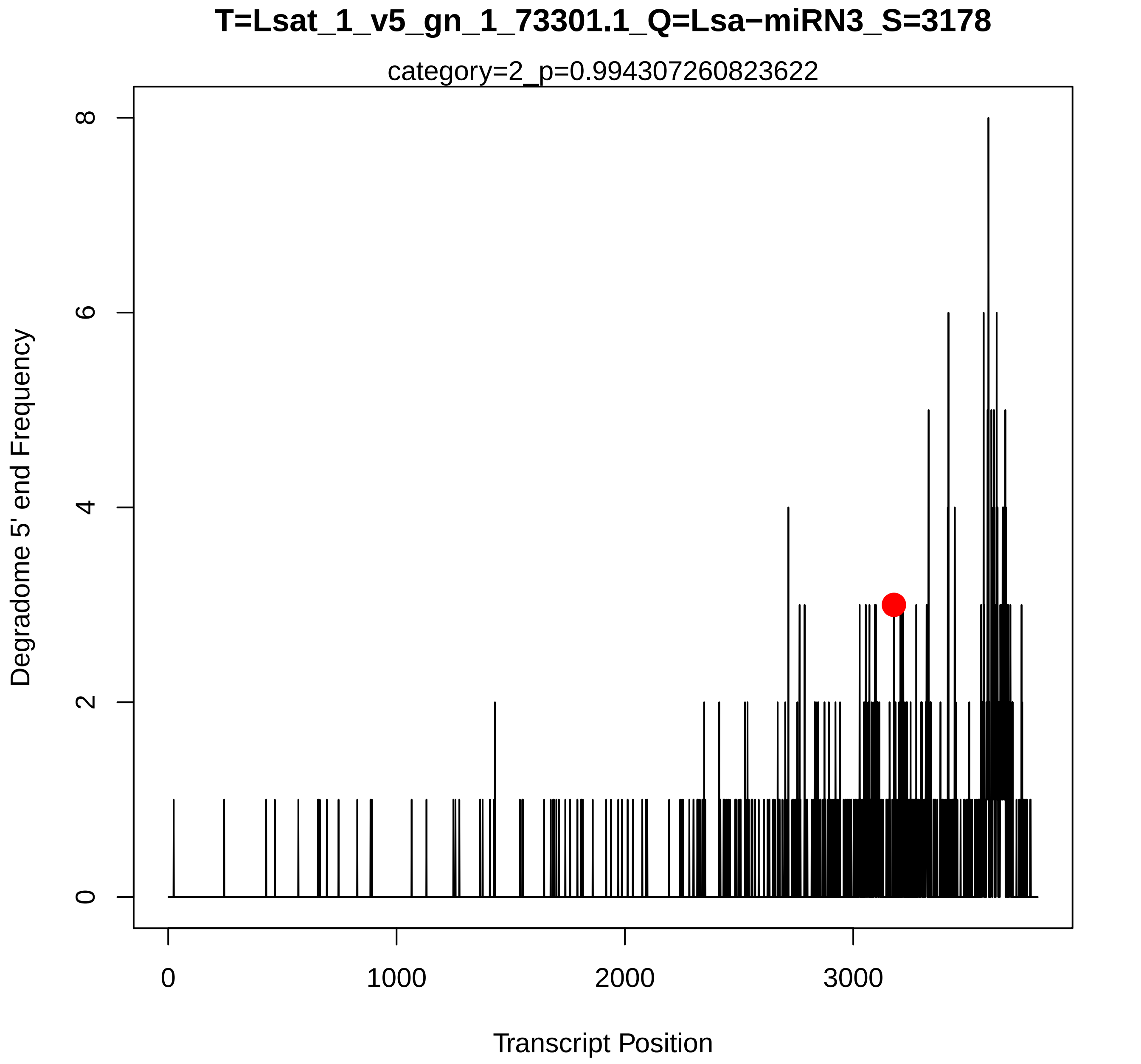
T=Lsat_1_v5_gn_1_73301.1_Q=Lsa−miRN3_S=3178
category=2_p=0.994307260823622
Degradome 5' end Frequency
0
2
4
6
8
0
1000
2000
3000
Transcript Position
